# Do Community Social Capital and Built Environment Associate With Homebound in Older Adults? The JAGES Niigata Study

**DOI:** 10.2188/jea.JE20200154

**Published:** 2022-06-05

**Authors:** Tomoko Tsubokawa, Yugo Shobugawa, Seitaro Iguchi, Tsubasa Suzuki, Michiko Watanabe, Reiko Saito, Katsunori Kondo

**Affiliations:** 1 Division of International Health (Public Health), Niigata University Graduate School of Medical and Dental Sciences, Niigata, Japan; 2 Faculty of Nursing (Public Health Nursing), Niigata Seiryo University, Niigata, Japan; 3 Department of Active Ageing (donated by Tokamachi city, Niigata Japan), Niigata University Graduate School of Medical and Dental Sciences, Niigata, Japan; 4 Department of Community Medicine, Niigata University Graduate School of Medical and Dental Sciences, Niigata, Japan; 5 Niigata Health Service Center, Niigata, Japan; 6 Center for Preventative Medical Sciences, Chiba University, Chiba, Japan; 7 National Center for Geriatrics and Gerontology, Aichi, Japan

**Keywords:** community social capital, neighborhood built environment, epidemiology, homebound, urban rural differences

## Abstract

**Background:**

Homebound status is one of the most important risk factors associated with functional decline and long-term care in older adults. Studies show that neighborhood built environment and community social capital may be related to homebound status. This study aimed to clarify the association between homebound status for community-dwelling older adults and community environment—including social capital and neighborhood built environment—in rural and urban areas.

**Methods:**

We surveyed people aged 65 years and older residing in three municipalities of Niigata Prefecture, Japan, who were not certified as requiring long-term care. The dependent variable was homebound status; explanatory variables were community-level social capital and neighborhood built environment. Covariates were age, sex, household, marital status, socioeconomic status, instrumental activities of daily living, the Geriatric Depression Scale-15, self-rated health, number of diseases under care, and individual social capital. The association between community social capital or neighborhood built environment and homebound status, stratified by rural/urban areas, was investigated using multilevel logistic regression analysis.

**Results:**

Among older adults (*n* = 18,099), the homebound status prevalence rate was 6.9% in rural areas and 4.2% in urban areas. The multilevel analysis showed that, in rural areas, fewer older adults were homebound in communities with higher civic participation and with suitable parks or pavements for walking and exercising. However, no significant association was found between community social capital or neighborhood built environment and homebound status for urban older adults.

**Conclusion:**

Community social capital and neighborhood built environment were significantly associated with homebound status in older adults in rural areas.

## INTRODUCTION

Although several concepts and definitions exist, the term *homebound*, as applied to older adults, refers to a situation in which the individual has few opportunities to leave the home.^1^^–^^4^ A study in Japan defined a person as homebound when their “frequency of going outdoors is less than once per week”.^5^ Many studies utilize this definition.^6^^–^^9^

Among older individuals, becoming homebound is thought to lead to an increased risk of mortality or need for long-term care.^6^^,^^7^^,^^10^^,^^11^ In Japan, measures were taken to prevent older adults from becoming homebound, but focused on individual factors (eg, instrumental activities of daily living [IADL] and physical capacity).^5^ Namely, a high-risk approach that focused on high risk individuals, such as those with physical functional declining, was the widely utilized intervention strategy. Recently, to prevent homebound status, there has been growing interest in the social interactions of community-dwelling older people.^12^ Moreover, the importance of community social capital (SC) in the local community has been drawing attention^13^; specifically, SC is an important social determinant of health and a topic of increasing interest in the social epidemiology and community health fields.^14^

Similarly, the neighborhood built environment is an important factor that influences behavior. For instance, people tend to prefer walking in environments that have parks and walking pavements.^15^^–^^18^ A study in a city that was severely affected by the 2011 Great East Japan Earthquake showed that having to walk long distances to reach a retail store might be a risk factor of homebound status among older adults.^17^ Thus, the literature suggests that the homebound status of community-dwelling older adults relates to both the social and physical environments. However, no prior study has analyzed the relationship between community level SC and homebound status. Additionally, few studies have analyzed the association between neighborhood built environment and the homebound status on older adults.^15^^,^^18^

Further, there are significant differences regarding the type of neighborhood built environment between rural and urban areas. Regarding the physical environment, the number of possible destinations (eg, restaurants, retail stores) in rural areas is lower than in urban areas. Regarding the social environment, social contact/connectedness is often higher in rural than in urban areas.^19^ Thus, the impact of the social and physical environments on homebound status might differ between rural and urban areas.

Thence, this study aimed to clarify the association between the homebound status on older adults and the community environment—including SC and neighborhood built environment—in rural and urban areas.

## METHODS

### Data

This study used cross-sectional data from the 2013 Japan Gerontological Evaluation Study (JAGES) Niigata survey.^12^ The project has accumulated data on older people over the age of 65 years who have not been certified as requiring long-term care. Research participants were recruited from Niigata City, Tokamachi City, and Aga Town. Niigata City is the prefectural capital and a city designated by ordinance with a population of approximately 0.8 million and a population density of 1115.2/km^2^. It is divided into urban, suburban resident, and countryside areas. Tokamachi City (population, 55,491 [as of 2015]; population density, 93.0/km^2^) and Aga Town (population, 11,946 [as of 2015]; population density, 12.3/km^2^) are located in mountainous regions and have urban and deep rural areas. Taken together, there is a rich variation in the environmental characteristics of the two cities and the town. Thus, we targeted these sites to conduct this research.

By stratified random sampling, we extracted 8,000 older people (4.9%) in Niigata City (from a total of 164,206 older people). The researchers conducted an inventory survey in Tokamachi City and Aga Town, targeting 15,730 and 4,192 older people, respectively.

The community unit of this study was set based on the school districts or living areas where the target people lived; in total, there were 57 communities in Niigata City, 19 in Tokamachi City, and 12 in Aga Town. The survey was conducted by the mail survey method in all municipalities. We targeted people who did not lack any sex, age, or resident community information.

### Outcome measure

The dependent variable was the homebound status, defined as going out of the house less than once a week.^5^^,^^9^ For the question “How often do you go out? (including farms/fields, neighbors’ homes, shopping, hospitals, etc),” there were six answer options: “4 or more times a week,” “2 to 3 times a week,” “Once a week,” “1 to 3 times/month,” “Several times/year,” and “None.” People was considered as under the homebound status if they answered “1 to 3 times/month,” “several times/year,” or “never.”

### Definition of rural or urban areas

Communities in a habitable area with a population density of at least 1,000 people/km^2^ or more were considered as urban; those under 1,000 people/km^2^ were considered as rural.^20^ The total population of each district was calculated using data from the 2010 National Census.

### Independent variables

The independent variables included two types of community level variables: Community SC and neighborhood built environment. We used the measurement index developed by Saito et al to assess community SC.^21^ It is a 11-item measurement with three indices/subscales: civic participation (5 items, hereinafter SC-CP), social cohesion (3 items, SC-SC), and reciprocity (3 items, hereinafter SC-RC). The SC-CP has questions on whether people participated in local meetings or the following group activities: volunteering, sports, hobbies, learning and education, and the passing down of experience. After calculating the percentage of those who participated in each activity on a community-by-community basis, the score in this component was calculated as: Percentage of volunteer group participants × 0.6 + percentage of sports group participants × 0.8 + percentage of hobbies group participants × 0.9 + percentage of learning and education group participants × 0.7 + percentage of passing down of experience group participants × 0.5.

The SC-SC has questions on community trust, mutual help, and community attachment. After calculating the percentage of those who answered positively (“I think so” or “I think”) on a community-by-community basis, the score in this component was calculated as: Percentage of positive community trust × 0.9 + percentage of positive mutual help × 0.8 + percentage of positive community attachment × 0.7.

The SC-RC has questions on whether participants provide/receive emotional/instrumental support. The percentage of those who provide/receive such social support on community-by-community basis, the score of this component was calculated as: Percentage of people who receive emotional support × 0.8 + percentage of people who provide emotional support × 0.7 + percentage of people who receive instrumental support × 0.6. All indices were dichotomized into high/low groups by the median value of the calculated indices.

Three types of neighborhood built environment were measured in this study: 1) Suitable parks or pavements for walking and exercising; 2) possible dangerous places or intersections that evoke risk for traffic accidents; and 3) grocery or mobile shops in which you can get fresh food. The question was: “How many facilities/places like these are present within approximately 1 kilometer from your house?” Participants chose one of five options: “many,” “some,” “few,” “none,” or “don’t know.” We calculated the percentage of participants who answered “many” or “some” in each community. Then, these percentages were dichotomized into high/low groups by median value.

### Covariates

We adjusted for the following possible confounding factors: age,^22^ sex,^22^ household, marital status,^22^ educational attainment,^23^ equivalized annual household income,^23^ categorized by depressive status using the Geriatric Depression Scale-15 (GDS-15) score,^22^^–^^27^ IADL,^24^ self-rated health (SRH),^22^^,^^28^ number of medical diseases under care or of sequelae,^22^ and individual SC.^13^

Age was categorized into five-year groups: 65–69, 70–74, 75–79, 80–84, and 85 or older; household into living alone and living with family members; marital status into married, widowed, divorced, single, and other; educational attainment into ≤9 years and ≥10 years; equivalized annual household income into <1 million yen, 1 million to <4 million yen, and ≥4 million yen; and GDS-15 score into no depression (≤4), mild depression (5–9), and depression (≥10).^28^ IADL was categorized using five items of instrumental independence corresponding to each IADL and comprising the subscales of the Tokyo Metropolitan Institute of Gerontology Index.^29^ Self-rated health (SRH) was classified as “very good/well” and “not very good/not good.” Number of medical diseases under care or of sequelae were categorized into “none,” “one,” “two,” and “three or more.”

For individual SC, we used the same indices for community SC: civic participation, social cohesion, and reciprocity. Civic participation was divided into five categories by the number of groups/activities people participated in: “none,” “one,” “two,” “three,” and “four or more.” Social cohesion into four categories by the number of positive responses to three questions about community trust, mutual aid, and community attachment: “none,” “one,” “two,” and “three.” Reciprocity comprised three items regarding community social support: Receiving and giving emotional support, and receiving instrumental support. Reciprocity was divided into four categories based on the number of items with responses other than “no one:” “none,” “one,” “two,” and “three.”

### Statistical analysis

To describe diverse prevalence rates for homebound status by community, we produced three graphs (for all 88 communities in rural and urban areas). To compare community characteristics between areas, we calculated the average value and standard deviation (SD) of the social and physical environmental indices and calculated the prevalence for homebound status for rural and urban areas. We applied the Welch’s *t* test to evaluate statistical differences. We also calculated the age- and sex-adjusted standardized prevalence rates for homebound status.

Participants’ demographic characteristics were divided and compared by rural and urban areas using a Chi-square test. The association between homebound status and the three community SC indices were analyzed through a multilevel logistic regression analysis stratified by rural and urban areas. The multilevel logistic regression analysis was performed in six steps: model 1 was a null model. In model 2, we included individual-level variables. In models 3, 4, and 5, we included variables in model 2 plus the SC-CP, SC-SC, or SC-RC scores, separately. In model 6, all three community SC indices were added to model 2 at the same time.

To examine the association between homebound status and three indices of neighborhood built environment, we applied another multilevel logistic regression analysis on the data stratified by rural and urban areas. The null model and model adjusted using individual-level variables are similar to models 1 and 2 in the analyses of community SC indices. In models 1 to 3, each neighborhood built environment index was included separately. In model 4, all neighborhood built environment indices were simultaneously included.

Moreover, we analyzed a combined model that included all six indices (three community SC and three neighborhood built environment) stratified by rural and urban areas. Additionally, we analyzed models stratified by sex to ensure that the same analyses would be done in the four stratified groups: Rural men, rural women, urban men, and urban women.

Statistical significance for all analyzes was set at *P* = 0.05. All analyses were performed using STATA 14 (Stata Corp, College Station, TX, USA).

### Ethical considerations

This study was conducted with ethical approval from the Institutional Review Board of Niigata University (approval numbers: 2015-1504, 2015-2045, and 2015-2046). Participants were informed that participation was voluntary and that returning the self-administered questionnaire would be interpreted as providing consent to participate.

## RESULTS

**Figure 1.**
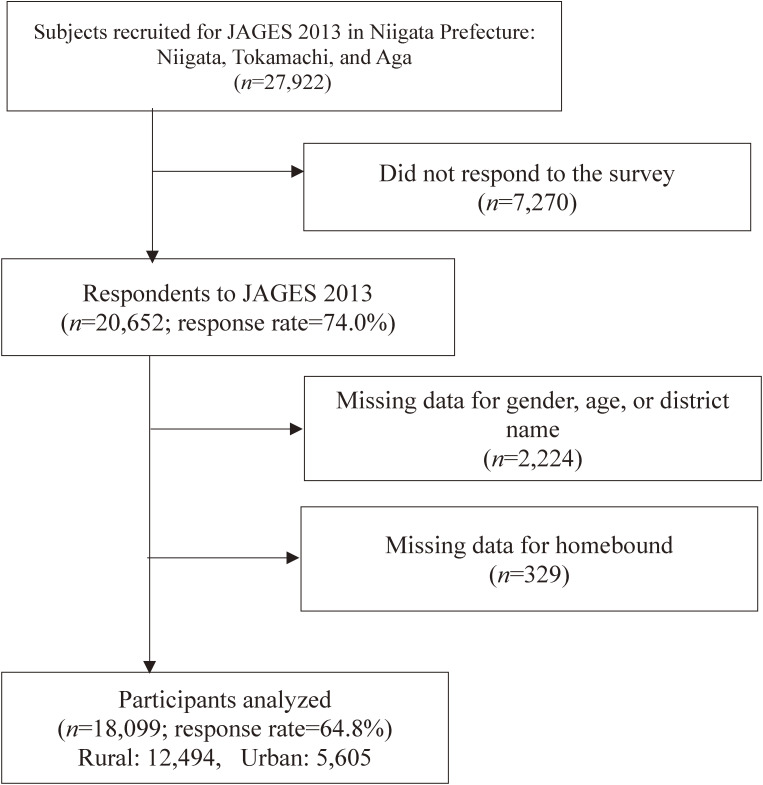
Flow chart of the sampling procedures

**Figure 2.**
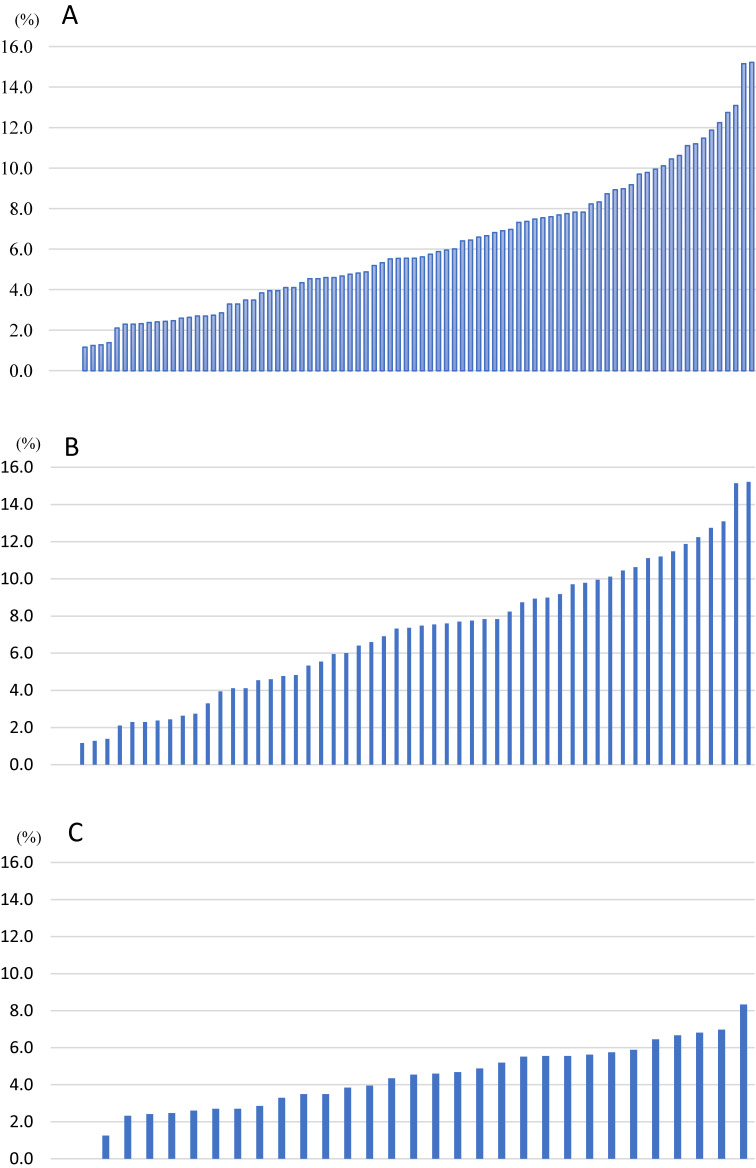
Prevalence rates of homebound status on older adults The prevalence rates in each community was represented from low to high prevalence among all analyzed 88 communities: (a) 52 communities in rural areas; (b) 32 in urban areas; and (c).

In total, 20,652 (74.0% of 27,922 subjects) responded to the survey. By excluding 2,224 questionnaires with no data on sex, age, or resident information in the first stage, and 329 with no homebound status data in the second stage, we extracted a final sample of 18,099 valid questionnaires (Valid response rate = 64.8%) (Figure 1). The number of valid respondents and response rate by municipality was 4,661 (58.3%) in Niigata City, 10,584 (67.3%) in Tokamachi City, and 2,854 (61.4%) in Aga Town. Average prevalence rate for homebound status was at its highest as 15.2% and at its lowest as 0% (Figure 2A). Similarly, average prevalence rates for homebound status were divided by rural and urban areas (Figure 2B and Figure 2C): In urban areas, prevalence rates for homebound status (ie, min 0.0% to max 8.3%) were less diverse than in rural areas.

### Comparison of community characteristics between rural and urban areas

Out of the 88 communities, 56 were classified as rural and 32 as urban. Table 1 shows community characteristics divided by rural and urban areas. As a result of calculating and comparing the average value and SD of the three community SC indices, SC-CP was significantly higher in urban (0.63; SD, 0.13) than in rural areas (0.48; SD, 0.15); SC-SC was significantly higher in urban (0.78; SD, 0.15) than in rural areas (0.65; SD, 0.14); and SC-RC had similar levels between rural (2.00; SD, 0.04) and urban areas (1.99; SD, 0.04).

**Table 1.** Community characteristics differences by rural and urban areas

	Rural (*n* = 56)	Urban (*n* = 32)	*P*-value^a^

	Mean (SD)	Mean (SD)	
**Community social capital**			
Civic participation	0.48 (0.15)	0.63 (0.13)	<0.001
Social cohesion	0.65 (0.14)	0.78 (0.15)	<0.001
Reciprocity	2.00 (0.04)	1.99 (0.04)	0.235
**Built environment**			
Suitable parks or pavements for walking and exercising, %	59.3 (13.3)	71.2 (10.2)	<0.001
Possible dangerous places or intersections that evoke risk of traffic accidents, %	51.4 (9.4)	60.3 (7.5)	<0.001
Grocery or mobile shops in which you can get fresh food, %	55.0 (17.9)	76.7 (10.5)	<0.001
**Prevalence of homebound status**			
Crude, %	6.9 (3.8)	4.2 (2.0)	<0.001
Age and sex adjusted, %	7.4	5.0	—

SD, standard deviation.

^a^Welch’s *t* test was applied.

For neighborhood built environment, there were many more communities with suitable parks or pavements for walking and exercising in urban (71.2%; SD, 10.2%) than in rural areas (59.3%; SD, 13.3%) (*P* < 0.001). There were many more communities with possible dangerous places or intersections that evoke risk for traffic accidents in urban (60.3%; SD, 7.5%) than in rural areas (51.4%; SD, 9.4%) (*P* < 0.001); and there were many more communities with grocery or mobile shops in which you can get fresh food in urban (76.7%; SD, 10.5%) than in rural areas (55.0%; SD, 17.9%) (*P* < 0.001).

Prevalence rate for homebound status was significantly higher in rural (6.9%; SD, 3.8%) than in urban areas (4.2%; SD, 2.0%). Age-adjusted prevalence rate for homebound status was 7.4% for rural and 5.0% for urban areas.

### Participants’ demographic characteristics by rural and urban areas

We analyzed participants’ demographic characteristics by rural and urban areas and used descriptive statistics (Table 2).

**Table 2.** Participants’ demographic characteristics by rural and urban areas

	Rural (*n* = 12,494)		Urban (*n* = 5,605)		*P* ^a^
	*n*	%	*n*	%	
**Sex**					
Men	5,690	45.5	2,560	45.7	0.869
Women	6,804	54.5	3,045	54.3	
**Age, years**					
65–69	3,264	26.1	1,529	27.3	<0.001
70–74	3,035	24.3	1,608	28.7	
75–79	2,805	22.5	1,264	22.6	
80–84	2,071	16.6	761	13.6	
≥85	1,319	10.6	443	7.9	
**Household**					
Living with others	10,028	80.3	4,598	82.0	0.005
Living alone	2,466	19.7	1,007	18.0	
**Marital status**					
Married	8,608	68.9	4,023	71.8	<0.001
Widowed, unmarried, other	3,169	25.4	1,419	25.3	
Missing data	717	5.7	163	2.9	
**Educational attainment, years**					
≤9	7,361	58.9	2,535	45.2	<0.001
≥10	4,830	38.7	2,973	53.0	
Missing data	303	2.4	97	1.7	
**Equivalized annual household income, million yen**					
<1	7,361	58.9	2,535	45.2	<0.001
1–3.99	4,830	38.7	2,973	53.0	
≥4	303	2.4	97	1.7	
Missing data	7,361	58.9	2,535	45.2	
**Geriatric depression scale-15 score**					
0–4	7,021	56.2	3,211	57.3	0.326
5–9	2,373	19.0	1,024	18.3	
≥10	735	5.9	347	6.2	
Missing data	2,365	18.9	1,023	18.3	
**Instrumental activities of daily living**					
0–4	9,477	75.9	4,432	79.1	<0.001
5	2,626	21.0	1,020	18.2	
Missing data	391	3.1	153	2.7	
**Self-rated health**					
Fair	9,806	78.5	4,479	79.9	0.092
Poor	2,281	18.3	959	17.1	
Missing data	407	3.3	167	3.0	
**Number of medical diseases under care or sequelae**					
0	2,873	23.0	1,218	21.7	<0.001
1	4,772	38.2	2,025	36.1	
2	2,900	23.2	1,403	25.0	
≥3	1,949	15.6	959	17.1	
**Individual social capital: Civic participation^b^**					
0	8,728	69.9	3,399	60.6	<0.001
1	2,137	17.1	1,137	20.3	
2	995	8.0	689	12.3	
≥3	634	5.1	380	6.8	
**Individual social capital: Social cohesion^c^**					
0	1,516	12.1	747	13.3	<0.001
1	1,744	14.0	892	15.9	
2	2,372	19.0	1,187	21.2	
3	6,862	54.9	2,779	49.6	
**Individual social capital: Reciprocity^d^**					
0	89	0.7	49	0.9	0.096
1	287	2.3	153	2.7	
2	826	6.6	338	6.0	
3	11,292	90.4	5,065	90.4	

^a^Chi-square test.

^b^Groups in which subjects participated more than once/month.

^c^Items with a positive response.

^d^Items with a response other than “no one”.

All following variables showed significant differences between rural and urban areas: regarding age, a higher proportion of older people was observed in rural than in urban areas. Living alone was more common in rural (19.7%) than in urban areas (18.0%). Regarding marital status, being married was more common in urban (71.8%) than in rural areas (68.9%). Regarding educational attainment, having ≥10 years of education was more common in urban (53.0%) than in rural areas (38.7%). Regarding equivalized household income, earning less than 1.00 million per year was more common in rural (58.9%) than in urban areas (45.2%). Regarding IADL, people with full scores (5) were more common in rural (21.0%) than in urban areas (18.2%). The percentage of people with “none” or “only one” medical diseases under care or sequelae was higher in rural than in urban areas (none: 23.0% vs 21.7%; only one: 38.2% vs 36.1%). Regarding the three individual-level SCs, there was a significantly higher proportion of people in the without much civic participation in rural (69.9%) than in urban areas (60.6%), and a significantly higher proportion of people with positive social cohesion (total score) in rural (54.9%) than in urban areas (49.6%).

### Results of multilevel logistic regression analyses

Table 3A and Table 3B show the results of multilevel logistic regression analyses for the association between community SC and homebound status by rural and urban areas. In the null model, community level variance was 0.149 in rural areas. However, there was no significant variation between communities in urban areas, in which community level variance was 4.7 × 10^−27^. The proportional changes in variance are shown at the bottom of Table 3A and Table 3B, which indicate community level variance owing to SC. SC-CP was significantly associated with homebound status (odds ratio [OR] 0.67; 95% confidence interval [CI], 0.51–0.88) in rural areas (model 3). SC-SC was marginally associated with homebound status (OR 0.74; 95% CI, 0.54–1.01) (model 4). There was no significant association between SC-RP and homebound status (model 5). In model 6, we observed similar associations for all three community SC indices. In urban areas, as shown in Table 3B, there was no significant association between homebound status and the community SC indices.

**Table 3A.** Result of multilevel logistic regression analysis to examine the association between community level SC and homebound status in rural areas

	Rural																						

	Model 1			Model 2				Model 3				Model 4				Model 5				Model 6			
					
	*n* = 12,494			*n* = 7,536				*n* = 7,536				*n* = 7,536				*n* = 7,536				*n* = 7,536			
	OR	95% CI	*P* ^a^	OR	95% CI		*P* ^a^	OR	95% CI		*P* ^a^	OR	95% CI		*P* ^a^	OR	95% CI		*P* ^a^	OR	95% CI		*P* ^a^
**Sex**																							
Men				Ref.				Ref.				Ref.				Ref.				Ref.			
Women				1.47	1.18	1.84	0.001	1.45	1.16	1.82	0.001	1.48	1.18	1.85	0.001	1.47	1.18	1.84	0.001	1.46	1.16	1.82	0.001
**Age, years**																							
65–69				1.00				1.00				1.00				1.00				1.00			
70–74				1.09	0.76	1.56	0.653	1.08	0.75	1.55	0.686	1.09	0.76	1.57	0.631	1.09	0.76	1.56	0.652	1.08	0.75	1.56	0.668
75–79				1.40	0.99	1.99	0.058	1.40	0.99	1.99	0.059	1.41	0.99	2.00	0.056	1.40	0.99	1.99	0.057	1.41	0.99	2.00	0.055
80–84				2.24	1.57	3.18	0.000	2.21	1.55	3.14	<0.001	2.24	1.57	3.19	<0.001	2.24	1.57	3.18	<0.001	2.22	1.56	3.15	<0.001
≥85				4.12	2.86	5.92	0.000	4.08	2.83	5.86	<0.001	4.12	2.86	5.93	<0.001	4.12	2.86	5.92	<0.001	4.09	2.84	5.89	<0.001
**Household**																							
Living with others				Ref.				Ref.				Ref.				Ref.				Ref.	Ref.		
Living alone				0.93	0.69	1.25	0.611	0.92	0.68	1.24	0.590	0.94	0.70	1.27	0.694	0.93	0.69	1.25	0.615	0.94	0.70	1.27	0.683
**Marital status**																							
Married				Ref.				Ref.				Ref.				Ref.				Ref.			
Widowed, unmarried, other				1.49	1.17	1.91	0.001	1.49	1.16	1.90	0.002	1.47	1.15	1.89	0.002	1.49	1.17	1.91	0.001	1.47	1.15	1.88	0.002
**Educational attainment, years**																							
≤9				Ref.				Ref.				Ref.				Ref.				Ref.			
≥10				0.84	0.66	1.08	0.173	0.84	0.66	1.07	0.155	0.86	0.67	1.09	0.216	0.84	0.66	1.08	0.175	0.85	0.67	1.09	0.201
**Equivalized annual household income, million yen**																							
<1				Ref.				Ref.				Ref.				Ref.				Ref.			
1–3.99				0.60	0.47	0.77	<0.001	0.61	0.48	0.77	<0.001	0.60	0.47	0.77	<0.001	0.60	0.47	0.77	<0.001	0.61	0.48	0.78	<0.001
≥4				0.64	0.41	1.00	0.049	0.65	0.42	1.01	0.054	0.65	0.42	1.01	0.053	0.64	0.41	1.00	0.048	0.66	0.42	1.02	0.060
**Geriatric depression scale-15 score**																							
0–4				Ref.				Ref.				Ref.				Ref.				Ref.			
5–9				1.60	1.27	2.03	<0.001	1.61	1.27	2.03	<0.001	1.61	1.27	2.03	<0.001	1.60	1.26	2.03	<0.001	1.61	1.28	2.04	<0.001
≥10				1.49	1.06	2.10	0.022	1.49	1.06	2.09	0.023	1.51	1.07	2.12	0.019	1.49	1.06	2.10	0.022	1.50	1.07	2.12	0.020
**Instrumental activities of daily living**																							
0–4				Ref.				Ref.				Ref.				Ref.				Ref.			
5				3.15	2.52	3.94	<0.001	3.16	2.53	3.95	<0.001	3.13	2.51	3.92	<0.001	3.15	2.52	3.94	<0.001	3.14	2.51	3.92	<0.001
**Self-rated health**																							
Fair				Ref.				Ref.				Ref.				Ref.				Ref.			
Poor				2.03	1.60	2.58	<0.001	2.05	1.61	2.60	<0.001	2.03	1.59	2.58	<0.000	2.03	1.60	2.58	<0.001	2.04	1.60	2.59	<0.001
**Number of medical diseases under care or sequelae**				Ref.				Ref.				Ref.				Ref.				Ref.			
0				Ref.				Ref.				Ref.				Ref.				Ref.			
1				1.04	0.76	1.41	0.824	1.03	0.76	1.40	0.864	1.04	0.76	1.41	0.815	1.04	0.76	1.41	0.824	1.03	0.76	1.40	0.848
2				0.98	0.70	1.37	0.920	0.98	0.70	1.37	0.908	1.00	0.71	1.39	0.993	0.98	0.70	1.37	0.921	1.00	0.71	1.39	0.989
≥3				0.91	0.63	1.30	0.588	0.90	0.63	1.28	0.548	0.92	0.64	1.32	0.657	0.91	0.63	1.30	0.591	0.91	0.64	1.31	0.617
**Individual social capital: Civic participation^b^**																							
0				Ref.				Ref.				Ref.				Ref.				Ref.			
1				0.34	0.23	0.51	<0.001	0.35	0.24	0.53	<0.001	0.34	0.23	0.52	<0.001	0.34	0.23	0.51	<0.001	0.35	0.24	0.53	<0.001
2				0.45	0.26	0.79	0.006	0.47	0.27	0.82	0.008	0.46	0.26	0.80	0.006	0.45	0.26	0.79	0.006	0.47	0.27	0.83	0.009
≥3				0.25	0.09	0.68	0.007	0.26	0.10	0.71	0.009	0.25	0.09	0.69	0.007	0.25	0.09	0.68	0.007	0.26	0.10	0.71	0.009
**Individual social capital: Social cohesion^c^**																							
0				Ref.				Ref.				Ref.				Ref.				Ref.			
1				0.66	0.46	0.96	0.030	0.66	0.46	0.96	0.030	0.67	0.46	0.97	0.034	0.66	0.46	0.96	0.031	0.67	0.46	0.97	0.033
2				0.68	0.48	0.96	0.029	0.67	0.48	0.95	0.025	0.68	0.48	0.96	0.029	0.68	0.48	0.96	0.030	0.68	0.48	0.95	0.025
3				0.51	0.37	0.70	<0.001	0.51	0.37	0.69	<0.001	0.50	0.37	0.69	<0.001	0.51	0.37	0.70	<0.001	0.50	0.36	0.68	<0.001
**Individual social capital: Reciprocity^d^**				Ref.				Ref.				Ref.				Ref.				Ref.			
0				Ref.				Ref.				Ref.				Ref.				Ref.			
1				1.11	0.40	3.11	0.836	1.12	0.40	3.11	0.831	1.10	0.39	3.08	0.851	1.11	0.40	3.11	0.836	1.11	0.40	3.09	0.840
2				0.97	0.38	2.45	0.943	0.95	0.37	2.39	0.907	0.94	0.37	2.38	0.897	0.97	0.38	2.45	0.943	0.92	0.37	2.33	0.867
3				0.74	0.30	1.80	0.503	0.73	0.30	1.78	0.487	0.72	0.29	1.75	0.463	0.74	0.30	1.80	0.501	0.71	0.29	1.74	0.459
**Community social capital indices**																							
Civic participation								0.67	0.51	0.88	0.004									0.68	0.52	0.89	0.006
Social cohesion												0.74	0.54	1.01	0.056					0.75	0.56	1.00	0.050
Reciprocity																1.02	0.79	1.31	0.890	0.94	0.73	1.21	0.620
					
Community-level variance (SE)	0.149	(0.065)		0.021	(0.040)			7.7 × 10^−29^	(4.6 × 10^−15^)			0.025	(0.047)			0.021	(0.041)			2.1 × 10^−28^	(1.1 × 10^−14^)		
PCV, %				86.0				14.0				−2.7				0.1				14.0			

CI, confidence interval; OR, odds ratio; PCV, proportional change in variance; SE, standard error.

^a^Chi-square test.

^b^Groups in which subjects participated more than once/month.

^c^Items with a positive response.

^d^Items with a response other than “no one”.

**Table 3B.** Result of multilevel logistic regression analysis to examine the association between community level SC and homebound status in urban areas

	Urban																						

	Model 1			Model 2				Model 3				Model 4				Model 5				Model 6			
					
	*n* = 5,605			*n* = 3,634				*n* = 3,634				*n* = 3,634				*n* = 3,634				*n* = 3,634			
	OR	95% CI	*P* ^a^	OR	95% CI		*P* ^a^	OR	95% CI		*P* ^a^	OR	95% CI		*P* ^a^	OR	95% CI		*P* ^a^	OR	95% CI		*P* ^a^
**Sex**																							
Men				Ref.				Ref.				Ref.				Ref.				Ref.			
Women				1.41	0.94	2.13	0.098	1.43	0.95	2.15	0.090	1.41	0.94	2.12	0.101	1.43	0.95	2.15	0.089	1.43	0.95	2.16	0.087
**Age, years**																							
65–69				Ref.				Ref.				Ref.				Ref.				Ref.			
70–74				0.58	0.29	1.16	0.124	0.59	0.30	1.17	0.129	0.58	0.29	1.16	0.124	0.58	0.29	1.16	0.123	0.58	0.29	1.16	0.125
75–79				1.37	0.75	2.48	0.304	1.39	0.77	2.52	0.277	1.37	0.75	2.48	0.302	1.37	0.76	2.48	0.299	1.40	0.77	2.53	0.271
80–84				2.28	1.25	4.16	0.007	2.36	1.29	4.30	0.005	2.27	1.24	4.13	0.008	2.29	1.26	4.17	0.007	2.30	1.26	4.21	0.007
≥85				4.49	2.39	8.43	<0.001	4.65	2.47	8.77	<0.001	4.50	2.40	8.45	<0.001	4.50	2.39	8.45	<0.001	4.66	2.47	8.80	<0.001
**Household**																							
Living with others				Ref.				Ref.				Ref.				Ref.				Ref.			
Living alone				1.25	0.72	2.17	0.423	1.26	0.72	2.18	0.418	1.25	0.72	2.17	0.423	1.25	0.72	2.17	0.422	1.26	0.72	2.18	0.416
**Marital status**																							
Married				Ref.				Ref.				Ref.				Ref.				Ref.			
Widowed, unmarried, other				0.98	0.61	1.56	0.923	0.97	0.61	1.55	0.897	0.98	0.61	1.57	0.936	0.98	0.61	1.56	0.918	0.98	0.61	1.57	0.940
**Educational attainment, years**																							
≤9				Ref.				Ref.				Ref.				Ref.				Ref.			
≥10				1.11	0.74	1.66	0.620	1.14	0.76	1.71	0.533	1.10	0.73	1.65	0.657	1.13	0.75	1.70	0.562	1.12	0.74	1.69	0.583
**Equivalized annual household income, million yen**																							
<1				Ref.				Ref.				Ref.				Ref.				Ref.			
1–3.99				1.08	0.72	1.63	0.707	1.09	0.72	1.64	0.697	1.08	0.72	1.63	0.708	1.08	0.72	1.63	0.708	1.08	0.72	1.64	0.703
≥4				1.32	0.74	2.36	0.348	1.30	0.73	2.33	0.377	1.32	0.74	2.37	0.344	1.32	0.74	2.36	0.350	1.32	0.73	2.36	0.358
**Geriatric depression scale-15 score**																							
0–4				Ref.				Ref.				Ref.				Ref.				Ref.			
5–9				1.44	0.94	2.20	0.091	1.43	0.94	2.19	0.095	1.44	0.94	2.21	0.090	1.43	0.94	2.19	0.096	1.43	0.94	2.19	0.097
≥10				1.52	0.82	2.78	0.180	1.52	0.83	2.80	0.174	1.53	0.83	2.81	0.174	1.50	0.82	2.75	0.193	1.54	0.84	2.83	0.167
**Instrumental activities of daily living**																							
0–4				Ref.				Ref.				Ref.				Ref.				Ref.			
5				5.79	3.86	8.69	<0.001	5.78	3.86	8.67	<0.001	5.82	3.88	8.73	<0.001	5.79	3.86	8.68	<0.001	5.87	3.91	8.81	<0.001
**Self-rated health**																							
fair				Ref.				Ref.				Ref.				Ref.				Ref.	.		
poor				1.77	1.16	2.71	0.009	1.77	1.15	2.70	0.009	1.77	1.16	2.72	0.008	1.76	1.15	2.70	0.009	1.78	1.16	2.73	0.008
**Number of medical diseases under care or sequelae**																							
0				Ref.				Ref.				Ref.				Ref.				Ref.			
1				1.18	0.65	2.14	0.583	1.17	0.65	2.13	0.595	1.18	0.65	2.14	0.580	1.18	0.65	2.14	0.588	1.17	0.65	2.13	0.598
2				1.30	0.70	2.40	0.411	1.30	0.70	2.42	0.399	1.29	0.70	2.40	0.415	1.30	0.70	2.41	0.404	1.30	0.70	2.40	0.412
≥3				1.12	0.57	2.19	0.743	1.13	0.58	2.21	0.722	1.11	0.57	2.18	0.760	1.13	0.57	2.21	0.728	1.11	0.56	2.17	0.768
**Individual social capital: Civic participation^b^**																							
0				Ref.				Ref.				Ref.				Ref.				Ref.			
1				0.25	0.12	0.53	<0.001	0.26	0.12	0.54	<0.001	0.25	0.12	0.53	<0.001	0.25	0.12	0.53	<0.001	0.25	0.12	0.54	<0.001
2				0.49	0.22	1.09	0.080	0.50	0.22	1.11	0.089	0.49	0.22	1.09	0.081	0.49	0.22	1.10	0.082	0.50	0.23	1.13	0.095
≥3				0.12	0.02	0.89	0.038	0.12	0.02	0.91	0.040	0.12	0.02	0.89	0.038	0.12	0.02	0.89	0.038	0.13	0.02	0.92	0.041
**Individual social capital: Social cohesion^c^**																							
0				Ref.				Ref.				Ref.				Ref.				Ref.			
1				1.51	0.74	3.12	0.260	1.52	0.74	3.13	0.256	1.52	0.74	3.13	0.257	1.51	0.73	3.10	0.265	1.53	0.74	3.15	0.251
2				1.59	0.80	3.17	0.189	1.54	0.77	3.09	0.221	1.61	0.80	3.21	0.181	1.55	0.78	3.10	0.215	1.55	0.77	3.11	0.217
3				1.75	0.94	3.25	0.077	1.72	0.92	3.19	0.088	1.78	0.95	3.31	0.071	1.70	0.91	3.16	0.095	1.74	0.93	3.25	0.082
**Individual social capital: Reciprocity^d^**																							
0				Ref.				Ref.				Ref.				Ref.				Ref.			
1				0.53	0.09	3.22	0.491	0.51	0.08	3.09	0.463	0.53	0.09	3.20	0.487	0.51	0.09	3.11	0.469	0.48	0.08	2.90	0.424
2				0.88	0.17	4.52	0.876	0.86	0.17	4.41	0.855	0.87	0.17	4.50	0.871	0.85	0.17	4.36	0.846	0.81	0.16	4.13	0.799
3				0.49	0.10	2.38	0.377	0.48	0.10	2.31	0.359	0.49	0.10	2.38	0.378	0.47	0.10	2.27	0.348	0.45	0.09	2.18	0.322
					
**Community social capital indices**																							
Civic participation								0.79	0.54	1.17	0.241									0.83	0.55	1.24	0.360
Social cohesion												1.09	0.73	1.62	0.675					1.33	0.83	2.13	0.231
Reciprocity																1.25	0.81	1.94	0.315	1.38	0.81	2.37	0.236
					
Community-level variance (SE)	4.7 × 10^−27^	(8.0 × 10^−15^)		1.0 × 10^−32^	(2.0 × 10^−17^)			3.7 × 10^−37^	(1.8 × 10^−19^)			1.3 × 10^−31^	(1.2 × 10^−16^)			2.4 × 10^−31^	(2.0 × 10^−16^)			5.0 × 10^−36^	(2.2 × 10^−18^)		
PCV, %				100.0				0.0002				−0.0025				−0.0049				0.0002			

CI, confidence interval; OR, odds ratio; PCV, proportional change in variance; SE, standard error.

^a^Chi-square test.

^b^Groups in which subjects participated more than once/month.

^c^Items with a positive response.

^d^Items with a response other than “no one”.

For the neighborhood built environment indices, only the presence of suitable parks or pavements for walking and exercising tended to be inversely associated with homebound status in rural areas (OR 0.72; 95% CI, 0.52–1.01) (Table 4A). In urban areas, we observed no significant association between neighborhood built environment indices and homebound status (Table 4B). In the models with the three community SC and the three neighborhood built environment indices, only civic participation in rural areas show a statistically significant association with homebound status (*P* = 0.015) (Table 5).

**Table 4A.** Result of multilevel logistic regression analysis to examine the association between neighborhood built environment and homebound status in rural areas

	Rural															
	
	Model 1 (*n* = 7,536)				Model 2 (*n* = 7,536)				Model 3 (*n* = 7,536)				Model 4 (*n* = 7,536)			
				
	OR	95% CI		*P* ^a^	OR	95% CI		*P* ^a^	OR	95% CI		*P* ^a^	OR	95% CI		*P* ^a^
**Sex**																
Men	Ref.				Ref.				Ref.				Ref.			
Women	1.48	1.18	1.85	0.001	1.47	1.17	1.84	0.001	1.48	1.18	1.84	0.001	1.48	1.18	1.84	0.001
**Age, years**																
65–69	Ref.				Ref.				Ref.				Ref.			
70–74	1.09	0.76	1.57	0.650	1.09	0.76	1.57	0.647	1.09	0.76	1.57	0.646	1.09	0.76	1.57	0.643
75–79	1.41	0.99	2.00	0.057	1.40	0.98	1.99	0.061	1.41	0.99	2.00	0.055	1.40	0.99	2.00	0.058
80–84	2.22	1.56	3.16	<0.001	2.23	1.57	3.17	<0.001	2.24	1.58	3.19	<0.001	2.22	1.56	3.15	<0.001
≥85	4.11	2.86	5.91	<0.001	4.11	2.85	5.91	<0.001	4.13	2.87	5.94	<0.001	4.11	2.85	5.91	<0.001
**Household**																
Living with others	Ref.				Ref.				Ref.				Ref.			
Living alone	0.93	0.69	1.26	0.640	0.92	0.68	1.24	0.584	0.92	0.68	1.25	0.603	0.92	0.68	1.25	0.610
**Marital status**																
Married	Ref.				Ref.				Ref.				Ref.			
Widowed, unmarried, other	1.48	1.15	1.89	0.002	1.49	1.17	1.91	0.001	1.49	1.17	1.91	0.001	1.48	1.16	1.90	0.002
**Educational attainment, years**																
≤9	Ref.				Ref.				Ref.				Ref.			
≥10	0.85	0.67	1.09	0.199	0.84	0.66	1.07	0.165	0.85	0.66	1.08	0.184	0.85	0.67	1.09	0.200
**Equivalized annual household income, million yen**																
<1	Ref.				Ref.				Ref.				Ref.			
1–3.99	0.61	0.48	0.78	<0.001	0.61	0.47	0.77	<0.001	0.60	0.47	0.77	<0.001	0.61	0.48	0.78	<0.001
≥4	0.65	0.42	1.00	0.052	0.65	0.42	1.01	0.053	0.64	0.41	1.00	0.048	0.65	0.42	1.01	0.056
**Geriatric depression scale-15 score**																
0–4	Ref.				Ref.				Ref.				Ref.			
5–9	1.60	1.27	2.03	<0.001	1.61	1.27	2.04	<0.001	1.60	1.27	2.03	<0.001	1.61	1.27	2.04	<0.001
≥10	1.50	1.07	2.12	0.020	1.50	1.06	2.11	0.021	1.49	1.06	2.11	0.021	1.51	1.07	2.12	0.019
**Instrumental activities of daily living**																
0–4	Ref.				Ref.				Ref.				Ref.			
5	3.15	2.52	3.94	<0.001	3.15	2.52	3.94	<0.001	3.14	2.51	3.92	<0.001	3.14	2.51	3.93	<0.001
**Self-rated health**																
fair	Ref.				Ref.				Ref.				Ref.			
poor	2.02	1.59	2.58	<0.001	2.03	1.60	2.59	<0.001	2.03	1.60	2.58	<0.001	2.03	1.59	2.58	<0.001
**Number of medical diseases under care or sequelae**																
0	Ref.				Ref.				Ref.				Ref.			
1	1.04	0.76	1.41	0.817	1.03	0.76	1.41	0.834	1.03	0.76	1.41	0.832	1.03	0.76	1.41	0.831
2	0.99	0.71	1.38	0.961	0.98	0.70	1.37	0.923	0.99	0.71	1.38	0.930	0.99	0.71	1.39	0.970
≥3	0.92	0.64	1.31	0.635	0.91	0.63	1.30	0.587	0.91	0.63	1.30	0.592	0.92	0.64	1.31	0.634
**Individual social capital: Civic participation^b^**																
0	Ref.				Ref.				Ref.				Ref.			
1	0.34	0.23	0.52	<0.001	0.34	0.23	0.52	<0.001	0.34	0.23	0.51	<0.001	0.34	0.23	0.52	<0.001
2	0.46	0.26	0.80	0.006	0.46	0.26	0.80	0.006	0.46	0.26	0.80	0.006	0.46	0.26	0.81	0.007
≥3	0.25	0.09	0.69	0.007	0.25	0.09	0.69	0.007	0.25	0.09	0.68	0.007	0.25	0.09	0.69	0.008
**Individual social capital: Social cohesion^c^**																
0	Ref.				Ref.				Ref.				Ref.			
1	0.67	0.46	0.97	0.033	0.67	0.46	0.97	0.033	0.67	0.46	0.97	0.032	0.67	0.46	0.98	0.037
2	0.68	0.48	0.96	0.027	0.68	0.48	0.96	0.029	0.69	0.49	0.97	0.032	0.68	0.48	0.96	0.028
3	0.51	0.37	0.69	<0.001	0.51	0.37	0.70	<0.001	0.51	0.37	0.70	<0.001	0.51	0.37	0.69	<0.001
**Individual social capital: Reciprocity^d^**																
0	Ref.				Ref.				Ref.				Ref.			
1	1.13	0.41	3.17	0.810	1.11	0.40	3.10	0.847	1.10	0.40	3.08	0.849	1.12	0.40	3.14	0.827
2	0.96	0.38	2.43	0.930	0.96	0.38	2.44	0.931	0.96	0.38	2.43	0.934	0.95	0.37	2.42	0.915
3	0.73	0.30	1.79	0.493	0.73	0.30	1.79	0.494	0.73	0.30	1.78	0.486	0.72	0.29	1.77	0.477
			
**Built environment**																
Suitable parks or pavements for walking and exercising	0.72	0.52	1.01	0.060									0.72	0.51	1.02	0.066
Possible dangerous places or intersections that evoke risk of traffic accidents					0.88	0.65	1.19	0.403					0.90	0.67	1.21	0.490
Grocery or mobile shops in which you can get fresh food									0.87	0.65	1.16	0.328	0.91	0.67	1.22	0.523
			
Community-level variance (SE)	0.018	(0.042)			0.033	(0.045)			0.022	(0.044)			0.028	(0.046)		
PCV, %	2.2				−8.3				−1.0				−4.4			

CI, confidence interval; OR, odds ratio; PCV, proportional change in variance; SE, standard error.

^a^Chi-square test.

^b^Groups in which subjects participated more than once/month.

^c^Items with a positive response.

^d^Items with a response other than “no one”.

**Table 4B.** Result of multilevel logistic regression analysis to examine the association between neighborhood built environment and homebound status in urban areas

	Urban															

	Model 1 (*n* = 3,634)				Model 2 (*n* = 3,634)				Model 3 (*n* = 3,634)				Model 4 (*n* = 3,634)			
			
	OR	95% CI		*P* ^a^	OR	95% CI		*P* ^a^	OR	95% CI		*P* ^a^	OR	95% CI		*P* ^a^
**Sex**																
Men	Ref.				Ref.				Ref.				Ref.			
Women	1.41	0.94	2.12	0.100	1.42	0.94	2.13	0.096	1.41	0.94	2.13	0.098	1.41	0.94	2.13	0.098
**Age, years**																
65–69	Ref.				Ref.				Ref.				Ref.			
70–74	0.59	0.29	1.16	0.126	0.59	0.29	1.16	0.127	0.58	0.29	1.16	0.125	0.59	0.30	1.17	0.128
75–79	1.38	0.76	2.50	0.291	1.37	0.76	2.48	0.301	1.37	0.75	2.48	0.303	1.38	0.76	2.49	0.293
80–84	2.29	1.26	4.18	0.007	2.34	1.28	4.27	0.006	2.28	1.25	4.16	0.007	2.33	1.27	4.26	0.006
≥85	4.53	2.41	8.52	<0.001	4.56	2.43	8.57	<0.001	4.49	2.39	8.43	<0.001	4.56	2.42	8.58	<0.001
**Household**																
Living with others	Ref.				Ref.				Ref.				Ref.			
Living alone	1.26	0.73	2.19	0.405	1.26	0.73	2.19	0.410	1.25	0.72	2.17	0.425	1.26	0.73	2.19	0.407
**Marital status**																
Married	Ref.				Ref.				Ref.				Ref.			
Widowed, unmarried, other	0.97	0.61	1.56	0.912	0.97	0.60	1.54	0.885	0.98	0.61	1.56	0.925	0.97	0.60	1.55	0.891
**Educational attainment, years**																
≤9	Ref.				Ref.				Ref.				Ref.			
≥10	1.10	0.73	1.65	0.645	1.13	0.75	1.70	0.563	1.11	0.74	1.66	0.624	1.12	0.74	1.68	0.597
**Equivalized annual household income, million yen**																
<1	Ref.				Ref.				Ref.				Ref.			
1–3.99	1.09	0.72	1.65	0.687	1.08	0.72	1.63	0.714	1.08	0.72	1.64	0.707	1.08	0.72	1.64	0.699
≥4	1.32	0.74	2.36	0.353	1.30	0.73	2.34	0.371	1.32	0.74	2.36	0.348	1.31	0.73	2.34	0.368
**Geriatric depression scale-15 score**																
0–4	Ref.				Ref.				Ref.				Ref.			
5–9	1.44	0.94	2.20	0.094	1.44	0.94	2.20	0.093	1.44	0.94	2.20	0.092	1.44	0.94	2.20	0.095
≥10	1.54	0.84	2.83	0.165	1.53	0.83	2.82	0.169	1.51	0.82	2.78	0.182	1.54	0.84	2.84	0.165
**Instrumental activities of daily living**																
0–4	Ref.				Ref.				Ref.				Ref.			
5	5.82	3.88	8.73	<0.001	5.75	3.83	8.63	<0.001	5.79	3.86	8.70	<0.001	5.78	3.85	8.67	<0.001
**Self-rated health**																
fair	Ref.				Ref.				Ref.				Ref.			
poor	1.78	1.16	2.73	0.008	1.77	1.16	2.71	0.009	1.77	1.15	2.71	0.009	1.78	1.16	2.72	0.008
**Number of medical diseases under care or sequelae**																
0	Ref.				Ref.				Ref.				Ref.			
1	1.17	0.65	2.13	0.598	1.17	0.65	2.13	0.600	1.18	0.65	2.14	0.583	1.17	0.65	2.12	0.605
2	1.29	0.69	2.39	0.422	1.29	0.70	2.40	0.413	1.30	0.70	2.40	0.410	1.29	0.70	2.39	0.419
≥3	1.10	0.56	2.16	0.776	1.12	0.57	2.20	0.733	1.12	0.57	2.19	0.743	1.11	0.57	2.18	0.755
**Individual social capital: Civic participation^b^**																
0	Ref.				Ref.				Ref.				Ref.			
1	0.25	0.12	0.53	<0.001	0.25	0.12	0.54	<0.001	0.25	0.12	0.53	<0.001	0.25	0.12	0.53	<0.001
2	0.49	0.22	1.10	0.083	0.49	0.22	1.10	0.085	0.49	0.22	1.09	0.080	0.49	0.22	1.10	0.085
≥3	0.12	0.02	0.90	0.039	0.12	0.02	0.90	0.039	0.12	0.02	0.89	0.038	0.12	0.02	0.90	0.039
**Individual social capital: Social cohesion^c^**																
0	Ref.				Ref.				Ref.				Ref.			
1	1.50	0.73	3.09	0.274	1.52	0.74	3.14	0.256	1.51	0.73	3.12	0.261	1.51	0.73	3.11	0.266
2	1.57	0.79	3.14	0.202	1.57	0.78	3.14	0.203	1.59	0.80	3.17	0.189	1.56	0.78	3.13	0.206
3	1.75	0.94	3.25	0.077	1.74	0.94	3.24	0.079	1.75	0.94	3.25	0.077	1.74	0.94	3.24	0.078
**Individual social capital: Reciprocity^d^**																
0	Ref.				Ref.				Ref.				Ref.			
1	0.52	0.09	3.19	0.482	0.51	0.08	3.10	0.467	0.53	0.09	3.22	0.491	0.51	0.08	3.11	0.467
2	0.89	0.17	4.59	0.887	0.86	0.17	4.44	0.862	0.88	0.17	4.52	0.876	0.87	0.17	4.50	0.872
3	0.50	0.10	2.44	0.392	0.49	0.10	2.36	0.371	0.49	0.10	2.38	0.377	0.49	0.10	2.40	0.382
**Built environment**																
Suitable parks or pavements for walking and exercising	1.17	0.81	1.71	0.400									1.10	0.71	1.71	0.670
Possible dangerous places or intersections that evoke risk of traffic accidents					0.83	0.57	1.22	0.350					0.87	0.56	1.36	0.552
Grocery or mobile shops in which you can get fresh food									1.02	0.41	2.54	0.967	1.03	0.40	2.64	0.949
			
Community-level variance (SE)	2.0 × 10^−33^	(1.0 × 10^−17^)			3.0 × 10^−33^	(3.0 × 10^−17^)			6.0 × 10^−31^	(5.0 × 10^−16^)			2.0 × 10^−34^	(2.0 × 10^−18^)		
PCV, %	0.0002				0.0002				−0.01				0.0002			

CI, confidence interval; OR, odds ratio; PCV, proportional change in variance; SE, standard error.

^a^Chi-square test.

^b^Groups in which subjects participated more than once/month.

^c^Items with a positive response.

^d^Items with a response other than “no one”.

**Table 5.** Result of multilevel logistic regression models combining all community level factors (ie, SC and neighborhood built environment) by rural and urban areas

	Rural				Urban			
	
	*n* = 7,536				*n* = 3,634			
	
	OR	95% CI		*P* ^a^	OR	95% CI		*P* ^a^
**Sex**								
Men	Ref.				Ref.			
Women	1.46	1.17	1.82	0.001	1.43	0.95	2.15	0.090
**Age, years**								
65–69	Ref.				Ref.			
70–74	1.08	0.75	1.56	0.666	0.58	0.29	1.16	0.125
75–79	1.41	0.99	2.00	0.054	1.38	0.76	2.51	0.285
80–84	2.21	1.56	3.15	<0.001	2.30	1.26	4.22	0.007
≥85	4.10	2.85	5.90	<0.001	4.62	2.44	8.72	<0.001
**Household**								
Living with others	Ref.				Ref.			
Living alone	0.94	0.69	1.26	0.671	1.27	0.73	2.21	0.398
**Marital status**								
Married	Ref.				Ref.			
Widowed, unmarried, other	1.47	1.15	1.88	0.002	0.98	0.61	1.57	0.921
**Educational attainment, years**								
≤9	Ref.				Ref.			
≥10	0.86	0.67	1.09	0.211	1.12	0.74	1.69	0.604
**Equivalized annual household income, million yen**								
<1	Ref.				Ref.			
1–3.99	0.61	0.48	0.78	<0.001	1.08	0.71	1.63	0.717
≥4	0.66	0.42	1.02	0.060	1.32	0.73	2.36	0.358
**Geriatric depression scale-15 score**								
0–4								
5–9	Ref.				Ref.			
≥10	1.62	1.28	2.05	<0.001	1.43	0.93	2.19	0.101
**Instrumental activities of daily living**	1.51	1.07	2.13	0.019	1.55	0.84	2.86	0.157
0–4								
5	Ref.				Ref.			
**Self-rated health**	3.13	2.50	3.91	<0.001	5.86	3.90	8.80	<0.001
fair								
poor	Ref.				Ref.			
**Number of medical diseases under care or sequelae**	2.03	1.60	2.59	<0.001	1.79	1.17	2.74	0.008
0								
1	Ref.				Ref.	Ref.		
2	1.03	0.76	1.40	0.847	1.16	0.64	2.11	0.617
≥3	1.00	0.72	1.40	0.997	1.28	0.69	2.38	0.432
**Individual social capital: Civic participation^b^**	0.91	0.64	1.31	0.625	1.09	0.56	2.15	0.796
0								
1	Ref.				Ref.			
2	0.35	0.23	0.53	<0.001	0.25	0.12	0.53	<0.001
≥3	0.47	0.27	0.83	0.009	0.50	0.23	1.13	0.094
**Individual social capital: Social cohesion^c^**	0.26	0.10	0.71	0.009	0.13	0.02	0.92	0.041
0								
1	Ref.				Ref.			
2	0.67	0.46	0.97	0.035	1.52	0.74	3.15	0.257
3	0.68	0.48	0.95	0.026	1.55	0.77	3.11	0.219
**Individual social capital: Reciprocity^d^**	0.50	0.36	0.68	<0.001	1.76	0.94	3.29	0.078
0								
1	Ref.				Ref.			
2	1.11	0.40	3.10	0.839	0.46	0.08	2.78	0.399
3	0.92	0.36	2.33	0.863	0.80	0.16	4.05	0.784
**Sex**	0.71	0.29	1.73	0.452	0.45	0.09	2.17	0.322
	
**Built environment**								
Suitable parks or pavements for walking and exercising	0.89	0.62	1.29	0.539	1.07	0.60	1.90	0.817
Possible dangerous places or intersections that evoke risk of traffic accidents	0.99	0.79	1.24	0.909	0.74	0.39	1.42	0.368
Grocery or mobile shops in which you can get fresh food	0.93	0.71	1.22	0.608	1.09	0.42	2.82	0.859
**Community social capital indices**								
Civic participation	0.70	0.52	0.93	0.015	1.09	0.54	2.20	0.806
Social cohesion	0.79	0.57	1.08	0.135	1.45	0.82	2.57	0.196
Reciprocity	0.93	0.72	1.21	0.603	1.64	0.86	3.12	0.130
	
Community-level variance (SE)	1.2 × 10^−30^	(5.8 × 10^−16^)			3.6 × 10^−33^	(9.4 × 10^−18^)		
PCV, %	14.1				0.0001			

CI, confidence interval; OR, odds ratio; PCV, proportional change in variance; SE, standard error.

^a^Chi-square test.

^b^Groups in which subjects participated more than once/month.

^c^Items with a positive response.

^d^Items with a response other than “no one”.

As a result of multilevel logistic regression analysis stratified by sex and area, SC-CP was significantly associated with homebound status in rural women (OR 0.54; 95% CI, 0.33–0.88) and SC-SC had a slightly significant association among rural women (OR 0.66; 95% CI, 0.41–1.08) (eTable 1A). The neighborhood built environment indices did not show any significant associations with homebound status (eTable 1B).

## DISCUSSION

This study investigated the associations between homebound status and community SC or neighborhood built environment in older adults in Niigata Prefecture, Japan. We divided communities by area (ie, rural and urban) and applied multilevel logistic regression analysis. After adjusting for individual factors, the results showed that community level civic participation—one of the indices in the community SC scale—and the presence of suitable parks or pavements for walking and exercising can prevent homebound status on older people.

### Prevalence rates of homebound status

Our results showed that the prevalence of homebound status on older adults was higher in rural than in urban areas, concurring with the literature.^3^^,^^6^^,^^30^^,^^31^ Compared with urban, rural areas offer fewer within-community destinations and group types to participate in (eg, hobby, sports, or volunteer groups), meaning fewer options/reasons for going out; accordingly, people in rural areas may find lesser opportunities/reasons to go out. Additionally, public transportation in rural areas of Japan is less frequent and less convenient.^32^ Nonetheless, we highlight a possible methodological limitation that justifies this between-group difference: Some rural area residents might understand the “going outdoors” phrase in our question as going out with a specific purpose (eg, shopping, medical consultation); thus, even if people in rural areas may regularly go out to do farm work or see their neighbors, they might not have considered this as “going outdoors.”

### Association between community SC and homebound status

Some factors may explain why we observed a diminished prevalence of homebound status on older adults with ample opportunities for civic participation in rural areas. Older adults’ participation in community activities may change their lives by: Empowering them through the development of community attachment; making them feel more safe and less anxious; recovering communication with neighbors, etc.^33^ Nonetheless, in rural areas, there are limited places to go and groups and activities to participate in, so rural older adults lack opportunities to go outdoors and into the community. Based on prior research, it may be that increasing the number of group activities and places to go/reasons to go outside in rural areas will reduce homebound status on older adults. Oppositely, in urban areas, older adults tend to have many opportunities to enter groups or to partake in activities, such as hobbies, sports, or volunteering work; they also have many reasons to go outside owing to the number of facilities at their disposal (eg, community center, gymnasium, grocery store).

We found some differences in the types of civic participation between people in rural and urban areas. Correlatively, Saito et al showed that hobby activities are more popular in urban than in rural areas.^31^ Tamakoshi showed that social and voluntary activities are more popular in rural than in urban areas.^34^ Our results showed that 19.1% of the rural sample and 28.4% of the urban sample participated in hobby groups; 12.9% of the rural and 18.2% of the urban in sports group; and 9.8% of the rural and 8.5% of the in volunteering (eTable 2). Thus, we need to pay attention to between-area differences regarding civic participation type, as such knowledge may allow for well-informed suggestions toward improvements in civic participation of older adults in rural and urban areas.

In the additional models stratified by sex, civic participation was significantly associated with older women in rural areas. In rural Japan, most women are homemakers and do not have enough opportunities for social participation. In such traditional contexts, women generally face hinderances to customary outings. Specifically, Japanese rural women tend to hesitate frequent home outings because they prefer not to be seen leaving the home by the neighbors, thereby being a cultural custom that obstructs their social participation.^35^ Under such circumstances, a civic participation activity may be a precious opportunity to allow these women to go outdoors and participate in social activities.

Moreover, we found no significant association between homebound status and the remaining two community SC indices in both rural and urban areas. Sato et al^20^ showed that the impact of SC-SC on self-rated health varied by urbanization level: It improved in urban areas, but not in rural ones. We could not compare such results directly with our findings owing to between-study differences regarding urbanization and different outcome settings. Notwithstanding, stakeholders should take between-area differences into account when considering the effects of SC on homebound status.

Our results also showed that individual-level social cohesion occurred more frequently in rural than in urban areas, whereas community level SC-SC (social cohesion) occurred more frequently in urban than in rural areas. This may be because a specific number of individuals with either high or low social cohesion scores were clustered in rural areas, also indicating higher variances at the community level. This may also be why we observed relatively lower average scores in community-level SC-SC in rural areas. To discuss SC-SC, we need to take into account the dark side of social capital.^36^ Sometimes, higher levels of social cohesion may have harmful effects on health owing to exclusive attitudes toward newcomers.

### Association between neighborhood built environment and homebound status

All three neighborhood built environment indices were lower in rural than in urban areas, indicating that rural people perceive that they do not have an appropriately neighborhood built environment (eg, not enough facilities within walking distance). Moreover, the presence of suitable parks or pavements for walking and exercising was significantly associated with diminished homebound status only in rural areas. Indeed, outdoor places in which people can go for walking and exercising may be potential destinations for older adults who do not have easy access to commercial or non-residential facilities.^32^ Specifically, the presence of parks or pavements explained 2.1% of the variance at the community level. Older adults surrounded by an environment that allows walking and exercising do not tend to be associated with the homebound status.

However, we found no significant association between grocery or mobile shops and homebound status. This result was not compatible with prior literature.^18^ Moreover, the presence of possible danger places or intersections for people who are walking, that evoke risk for traffic accidents, was not associated with homebound status in rural areas. This may be explained by the lack of traffic accidents in rural areas owing to limited traffic.

By analyzing the model with all community SC and neighborhood built environment indices, only community SC-CP was significantly associated with homebound status in rural areas. Thus, the effect of the presence of suitable parks or pavements disappeared in this model; this may be because there may have been a degree of correlation between social participation and the presence of such parks/pavements (correlation coefficient = 0.57). In urban areas, we observed no significant associations between neighborhood built environment indices and homebound status on older adults. One of the reasons for this lack of correlation may relate to diminished variances in urban communities in the first place. In other words, there may be poor environmental variation in urban rather than rural areas.

### Strengths and limitations

We acknowledge three strengths in this study. First, we placed homebound status as an outcome variable to evaluate its correlation with community level factors. The homebound status is deemed as a visible index because family members and neighbors can recognize it even at its early stages. Second, our study was conducted in the Niigata Prefecture, which has both urban areas typical to Japan and deep mountainous rural areas; this allowed for us to compare between-area differences in the same prefecture. Third, we applied a multilevel logistic regression analysis to consider not only participants’ individual characteristics but also community-level SC.

However, our study also has limitations. First, our results are limited to data from only three municipalities in a single prefecture (Niigata), so its representability is hindered. Second, there may be sample bias in our study results because, generally, 64% of our study participants who responded to the questionnaire were healthier than those who did not respond. Third, as remarked earlier, some Japanese rural residents might understand the question about “going outdoors” as referring to activities such as shopping or medical consultations, which have specific purposes; thus, they may not considered their daily farm work or visits to their neighbors as “going outdoors.”

We propose the following suggestions for future studies: first, longitudinal research is warranted to clarify causal relationships between being homebound and community social capital. Second, a similar analysis is needed using data from multiple prefectures with wide variations, as variety in the data is relatively limited within a single prefecture.

### Conclusion

Using multilevel analysis, our results indicated that there was a negative association between homebound status on rural older adults and the lack of community level civic participation and of suitable parks/pavements. Although these results were relevant only in rural areas, our study provides evidence that appropriately built environments in the neighborhood and community level SC may reduce homebound status; this is indicative of an effective public strategy that may be used by relevant stakeholders interested in improving Japanese older adults’ health and active ageing. Moreover, our results suggest the need to consider differences between rural and urban areas when developing intervention strategies to be applied in specific communities. Concluding, community-level improvements in SC and in the neighborhood built environment can promote active ageing in rural areas.

## APPENDIX A. SUPPLEMENTARY DATA

eTable 1A. Result of the multi-level logistic regression analysis to examine the association between community level SC and homebound status stratified by rural/urban areas and sex

eTable 1B. Result of the multi-level logistic regression analysis to examine the association between community level neighborhood built environment and homebound status stratified by rural/urban areas and sex

eTable 2. Types of civic participation by rural and urban areas
