# Supplementary material for: Do Community Social Capital and Built Environment Associate With Homebound in Older Adults? The JAGES Niigata Study
Source: J Epidemiol. 2022 Jun 5;32(6):254–69. doi: 10.2188/jea.JE20200154 (PMC9086311; doi:10.2188/jea.JE20200154)
Supplement: Supplementary file 1 [file je-32-254-s001.pdf]

eTable 1A. Result of the multi-level logistic regression analysis to examine the association between community level SC and homebound status stratified by rural/urban areas and sex

	Rural								Urban							
	Male (n=3,919)				Female (n=3,617)				Male (n=1,865)				Female (n=1,587)			
	OR	95% CI		P^a	OR	95% CI		P^a	OR	95% CI		P^a	OR	95% CI		P^a
**Community social capital indices**																
Civic participation	0.82	0.52	1.29	.381	0.54	0.33	0.88	.013	0.71	0.40	1.26	.239	0.95	0.52	1.74	.859
Social cohesion	0.72	0.44	1.17	.182	0.66	0.41	1.08	.098	1.19	0.64	2.23	.584	1.53	0.73	3.23	.264
Reciprocity	0.80	0.52	1.23	.314	1.05	0.70	1.56	.829	1.01	0.48	2.12	.979	1.79	0.79	4.05	.162
Community-level variance (SE)	0.08	(0.17)			0.10	(0.09)			2.0×10^{-33}	(3.4×10^{-17})			3.0×10^{-32}	(1.6×10^{-16})		
PCV, %	34.2				0.002				-2.2				-0.003			

CI, confidence interval; OR, odds ratio; PCV, proportional change in variance; SC, social capital; SE, standard error.

Age, household, marital status, educational attainment, equivalized annual household income, geriatric depression scale-15 score, instrumental activities of daily living, self-rated heath, number of medical diseases under care, individual social capital (civic participation, social cohesion, and reciprocity) were adjusted in all models.

[a] Chi-square test.

eTable 1B. Result of the multi-level logistic regression analysis to examine the association between community level neighborhood built environment and homebound status stratified by rural/urban areas and sex

	Rural								Urban							
	Male (n=3,919)				Female (n=3,617)				Male (n=1,865)				Female (n=1,587)			
	OR	95% CI		P^a	OR	95% CI		P^a	OR	95% CI		P^a	OR	95% CI		P^a
**Built environment**																
Suitable parks or pavements for walking and exercising	0.79	0.48	1.31	.367	0.65	0.39	1.07	.092	0.93	0.50	1.74	.828	1.43	0.74	2.76	.288
Possible dangerous places or intersections that evoke risk of traffic accidents	0.81	0.54	1.21	.299	0.91	0.58	1.43	.682	0.75	0.40	1.41	.373	1.05	0.54	2.02	.895
Grocery or mobile shops in which you can get fresh food	0.63	0.39	1.03	.065	1.16	0.74	1.82	.508	0.78	0.21	2.82	.699	1.53	0.38	6.14	.548
Community-level variance (SE)	0.04	0.12			0.10	0.09			(6.8×10^{-32})	(7.0×10^{-17})			(2.4×10^{-33})	(1.1×10^{-17})		
PCV, %	93.8				-10.2				-0.017				0.0005			

CI, confidence interval; OR, odds ratio; PCV, proportional change in variance; SE, standard error.
Age, household, marital status, education, equivalized annual household income, geriatric depression scale-15 score, instrumental activities of daily living, self-rated heath, number of medical diseases under care, individual social capital (civic participation, social cohesion, and reciprocity) were adjusted in all models.
[a] Chi-square test.

eTable 2. Types of civic participation by rural and urban areas

	Rural (n=56)	Urban (n=32)	*P*-value [a]
	Mean (SD)	Mean (SD)	
Civic participation			
Local meetings or group activities:			
Hobbies	19.1 (7.9)	28.4 (7.8)	<0.001
Sports	12.9 (4.8)	18.2 (4.6)	<0.001
Volunteers	9.8 (3.7)	8.5 (3.4)	0.111
Learning and education	4.6 (2.3)	6.9 (3.6)	0.002
The passing down of experience	3.2 (1.6)	4.7 (2.3)	0.001

SD, standard deviation.

[a] Welch's *t* test was applied.
